# The Major Surface-Associated Saccharides of *Klebsiella pneumoniae* Contribute to Host Cell Association

**DOI:** 10.1371/journal.pone.0003817

**Published:** 2008-11-26

**Authors:** Abigail Clements, Fabien Gaboriaud, Jérôme F. L. Duval, Jacinta L. Farn, Adam W. Jenney, Trevor Lithgow, Odilia L. C. Wijburg, Elizabeth L. Hartland, Richard A. Strugnell

**Affiliations:** 1 Australian Bacterial Pathogenesis Program, The University of Melbourne, Parkville, Victoria, Australia; 2 Co-operative Research Centre for Vaccine Technology, Department of Microbiology & Immunology, The University of Melbourne, Parkville, Victoria, Australia; 3 Laboratory of Physical Chemistry and Microbiology for the Environment, Nancy University, CNRS, Villers-les-Nancy, France; 4 Laboratory Environment and Mineral Processing, Nancy University, CNRS, Vandoeuvre-lès-Nancy, France; 5 Department of Biochemistry and Molecular Biology, Bio21 Institute, The University of Melbourne, Parkville, Victoria, Australia; Columbia University, United States of America

## Abstract

Analysing the pathogenic mechanisms of a bacterium requires an understanding of the composition of the bacterial cell surface. The bacterial surface provides the first barrier against innate immune mechanisms as well as mediating attachment to cells/surfaces to resist clearance. We utilised a series of *Klebsiella pneumoniae* mutants in which the two major polysaccharide layers, capsule and lipopolysaccharide (LPS), were absent or truncated, to investigate the ability of these layers to protect against innate immune mechanisms and to associate with eukaryotic cells. The capsule alone was found to be essential for resistance to complement mediated killing while both capsule and LPS were involved in cell-association, albeit through different mechanisms. The capsule impeded cell-association while the LPS saccharides increased cell-association in a non-specific manner. The electrohydrodynamic characteristics of the strains suggested the differing interaction of each bacterial strain with eukaryotic cells could be partly explained by the charge density displayed by the outermost polysaccharide layer. This highlights the importance of considering not only specific adhesin:ligand interactions commonly studied in adherence assays but also the initial non-specific interactions governed largely by the electrostatic interaction forces.

## Introduction

The physicochemical composition of the bacterial cell surface is an important factor in bacterial pathogenicity, since it is this surface that first interacts with soluble factors (eg. the humoral immune system) and the extracellular matrices of the infected host tissues and cells. Bacteria have evolved cell surface characteristics arising from carbohydrate and protein charge and composition under pressures exerted by their specific host environment. For example, many bacteria display a polysaccharide ‘coat’, either as a discrete layer of exopolysaccharide described as a capsule, or as a loosely attached slime layer. Bacterial capsules may include hyaluronic acid (eg. group A Streptococcus [1]) or sialic acid (eg. *Neisseria meningitidis* [2]) residues which can mimic human cell surfaces and thus reduce detection by the immune system. Polysaccharide capsules may also potentially favour the attachment to surfaces and the formation of biofilms through non-specific forces such as electrostatic and van der Waals forces and/or specific ligand-receptor interactions [3]–[5]. Capsules may also provide a measure of resistance to dessication in the environment [6].

Among the exopolysaccharides produced by gram-negative bacteria is lipopolysaccharide (LPS) comprising three distinct sections; lipid A, core and O-antigen (O-Ag) polysaccharide. The lipid A anchors the LPS molecule into the outer membrane and is also an endotoxin, stimulating the immune system through agonism of Toll-like receptor 4 (TLR4) which is present on macrophages, dendritic cells and other cell types. Agonism of TLR4 leads to intracellular signalling through MyD88-dependent and independent processes, inducing NF-kB mediated production of cytokines [7]. The core region of LPS, which links the O-Ag onto the lipid A molecule, contains a small number of mono-, di- or oligosaccharides (including 2–3 3-deoxy-D-manno-octulosonic acid (KDO) residues) and is negatively charged usually due to phosphate substitutions. Finally, the O-Ag consists of a variable number of saccharides repeated to form a polysaccharide layer that extends up to 30 nm into the surrounding media [8], [9].

In this study we investigated the cell surface properties of *Klebsiella pneumoniae*, a bacterium ubiquitous in nature, being present in the soil, surface water and often as normal gastrointestinal flora [10]–[12]. *K. pneumoniae* is also a human pathogen and is found with increasing frequency as a cause of hospital acquired infection, especially in intensive care and neonatal wards [13]–[15]. This bacterium possesses surface saccharides in the form of LPS and capsule, both of which have been linked with the virulence of *K. pneumoniae* in humans. Over 90% of clinical isolates from Denmark, Spain and the US were found to be O-Ag positive, of which O1 was the most common serotype [16] and between 70 and 80% of clinical isolates from Australia and Taiwan were capsule typeable (untypeable strains may be unencapsulated or of a novel capsule type) [17], [18].

The strain utilised in this study (B5055) is of the K2:O1 serotype. The K2 serotype repeat unit structure has been determined as [19]:
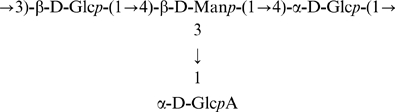



And the O1 LPS serotype:
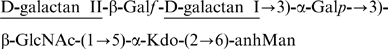
where D-galactan I [→3)-α-Gal*p*-(1→3)-β-Gal*f*-(1], and D-galactan II [→3)-β-Gal*p*-(1→3)-α-Gal*p*-(1], are repeating units [20].

Unlike many *Enterobacteriaceae*, the *K. pneumoniae* LPS core is not phosphorylated and the negative charge of the core is instead attributed to the presence of galacturonic acid (GalA) and KDO residues. Therefore investigations of the function of LPS in the commonly studied *Enterobacteriaceae* (eg. *E. coli* and *Salmonella sp*.) should not be extrapolated to *K. pneumoniae* without experimental confirmation.

In this study we generated mutations in *wzb/c* and in *waaF* of *K. pneumoniae* strain B5055 in order to study the contribution of the capsule and LPS saccharides to the surface structure and function of *K. pneumoniae.* We then examined the biological and physicochemical consequences of these changes to virulence. The results suggested that while capsular polysaccharides were essential for resisting innate immune mechanisms, the saccharides present in LPS may play a role in maintaining a neutral surface charge to allow association with the host cell to occur.

## Results

### Characterisation of saccharide mutants

**Figure 1:**
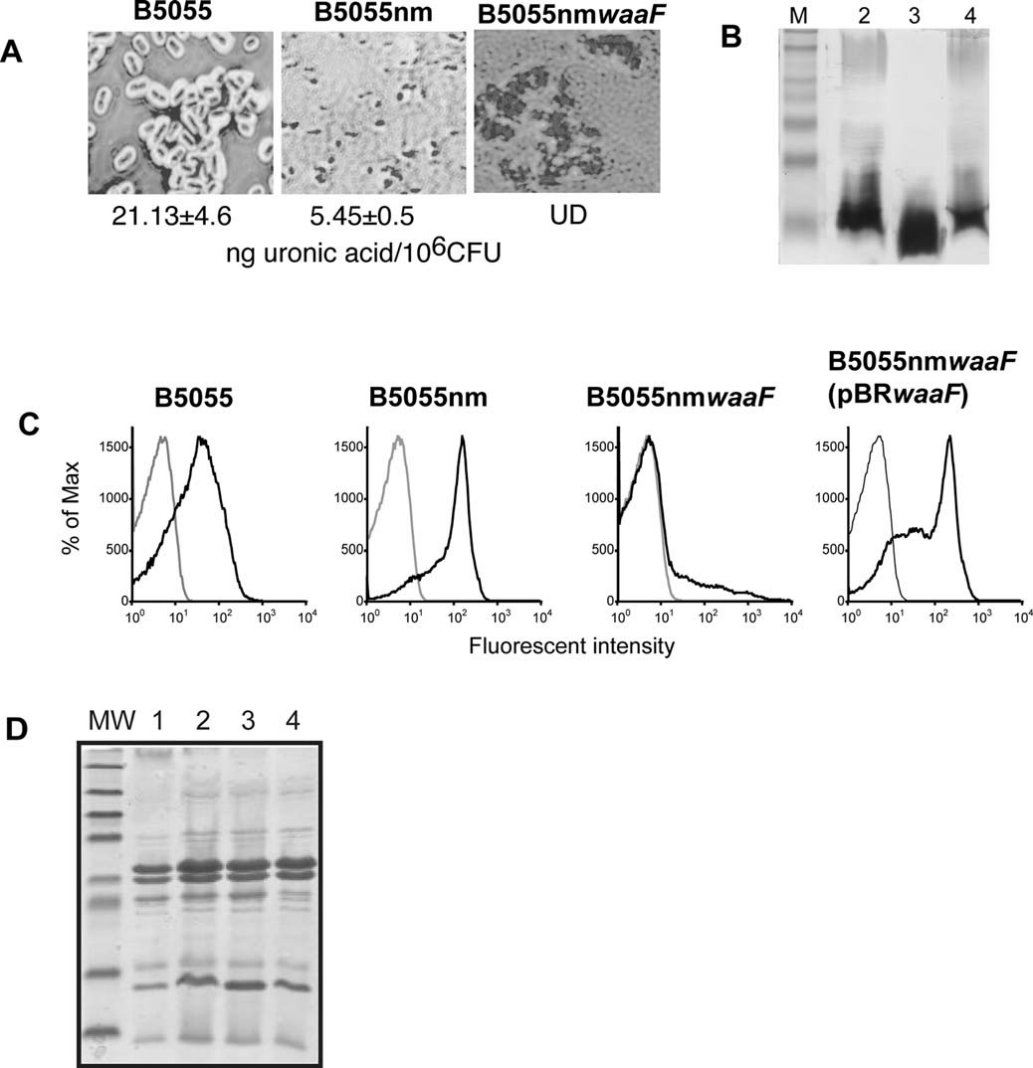
Characterisation of the surface layers of *K. pneumoniae* mutant strains. A) The capsule was visualised by Maneval's stain and quantified by uronic acid assay. UD = undetectable. B) LPS preparations were separated by SDS-PAGE on a 15% polyacrylamide gel and visualised by modified silver staining. C) Fluorescent activated cell sorting using mAb C13 specific for the O1-Ag of LPS. Only single cell populations (determined from cells stained with secondary antibody alone) were included in fluorescence analsyis. D) OMP preparations from mid-log cultures separated by 12.5% SDS-PAGE and visualised by Coomassie Brilliant Blue staining. Lanes are (1) B5055, (2) B5055nm, (3) B5055nm*waaF* and (4) B5055nm*waaF*+pBR*waaF*.

To assess the contribution of saccharides to *K. pneumoniae* virulence, mutations in *wzb-c* or *waaF* were successfully created in *K. pneumoniae* B5055 to produce an unencapsulated mutant (Δ*wzb-c*, B5055nm) and an unencapsulated, “deep-rough” LPS mutant (Δ*waaF*, B5055nm*waaF*). B5055*waaF* was also created but this strain appeared phenotypically unencapsulated; recent evidence indicates that “deep-rough” LPS mutants are unable to retain cell-associated capsule [21], [22] and therefore this strain was not used for *in vitro* assays. B5055, B5055nm and B5055nm*waaF* were characterised for the presence of capsule by Maneval's staining and uronic acid assays, and for LPS O-Ag by silver staining and immunofluorescence with an O1-Ag specific mAb (Fig. 1). The capsule was visualised in preparations of the wild type B5055, but not in B5055nm and B5055nm*waaF* (Fig. 1a); the amount of uronic acid, present as glucuronic acid in the capsule and galacturonic acid in the LPS, was reduced at least 10-fold for both mutant strains.

Proteinase K-digested whole cell lysates were separated by SDS-PAGE and LPS visualised by modified silver staining [23]. LPS O-Ag was observed in wild-type B5055 and unencapsulated B5055nm preparations, but not in the double mutant B5055nm*waaF* (Fig. 1b). The only proteinase K-resistant structure seen in this strain appeared to be the lipid A/core molecule, which migrated faster in SDS-PAGE than wild-type core, following the loss of the majority of core saccharides. Fluorescent microscopy and fluorescent activated cell sorter (FACS, Fig. 1c) analyses using a monoclonal antibody (mAb) previously determined to be specific to the O1-Ag of *K. pneumoniae* (designated mAb C13) indicated that both B5055 and B5055nm expressed the O1-Ag on the cell surface while B5055nm*waaF* showed no reactivity with this antibody indicating the O1-Ag is not expressed on the cell surface of this strain. FACS and microscopic analysis both indicated agglutination of the O-Ag positive, capsule negative strains (B5055nm and B5055nm*waaF* (pBR*waaF*)) with mAb C13. As a result FACS fluorescence analysis was restricted to single cells as gated in the no primary antibody controls for each bacterial strain. Growth rates of the three strains were compared in Luria broth and Dulbecco's Modified Eagle Medium (DMEM supplemented with L-glutamine and 10% FCS) and found to be equivalent (data not shown).

Outer membrane proteins (OMPs) were purified from each strain and compared by 1D SDS-PAGE (Fig. 1d). OMPs prepared from mid-log cultures showed identical banding patterns for all strains. Previous studies have found trimerisation of some OMPs (such as OmpF and C) to be reduced in deep rough LPS mutants [24]. While this may be occurring in B5055nm*waaF* the identical protein profile suggests this is not leading to a loss of any proteins (including potential adhesins) in the outer membrane.

### Resistance to innate immune mechanisms

The exopolysaccharide capsule is important in resistance to phagocytosis and/or complement-mediated lysis of *K. pneumoniae* [25], [26], while the O-Ag of LPS has been implicated in resistance to complement-mediated lysis [27], [28]. However, differing results for various strains has led to some doubt over these designations and we therefore analysed resistance of B5055, B5055nm and B5055nm*waaF* to these innate defence mechanisms.

Complement-mediated lysis assays were conducted both in the presence and absence of tetracycline (a bacteriostatic antibiotic) in order to determine whether complement factors in the serum were bactericidal, rather than bacteriostatic. The viable counts of unencapsulated mutants (ie. B5055nm, B5055nm*waaF* and B5055nm*waaF* (pBR*waaF*)) were all reduced by ∼3 logs within the initial 30 minute incubation indicative of killing by complement factors present in human serum (Table 1), suggesting that capsule is necessary for resistance to complement-mediated lysis. With FACS analysis having shown the O1-Ag is present on the bacterial cell surface of B5055nm, the inability of this strain to resist killing by complement factors suggested that the O1-Ag of LPS was unable to protect the bacterial membrane from killing by complement-mediated effectors. Whole blood phagocytosis assays [29] showed the same pattern as the complement-mediated lysis assays, suggesting that complement killing is an important innate immune mechanism in controlling septicemic *K. pneumoniae*.

**Table 1:** Resistance of *K. pneumoniae* strains to complement mediated lysis.

Strain	% survival (of inoculum) 60 min after incubation with sera
B5055	142
B5055+HTS*	105
B5055nm	0.0021
B5055nm+HTS*	107
B5055nmwaaF	0.0018
B5055nmwaaF+HTS*	106
B5055nmwaaF+pBR-waaF	0.012
B5055nmwaaF+pBR-waaF+HTS*	50

* HTS = Heat treated sera. Serum complement factors were inactivated by heating at 56°C for 30 min.

Note: A bacteriostatic antibiotic - tetracycline (25 µg/ml) - was added to samples to inhibit growth and hence allow direct measurement of bacterial killing by complement factors.

Figures are the average of duplicate samples, and the assay has been repeated in the presence and absence of tetracycline.

### Cell-association properties

Another important process in the establishment of a bacterial infection is the ability of the bacteria to associate with host cells, initially through surface adhesion [30]. Cell association is important simply to resist clearance (eg association with lung epithelial cells allows resistance of mucocilliary clearance), and thus facilitates colonisation and then pathology. The *K. pneumoniae* capsule has previously been implicated in impeding adherence to cells by physically shielding short adhesins, negating their function [31], [32]. We investigated the ability of our bacterial strains to associate with a mouse macrophage-like cell line (RAW264.7), and a human lung epithelial cell line (A549).

**Figure 2:**
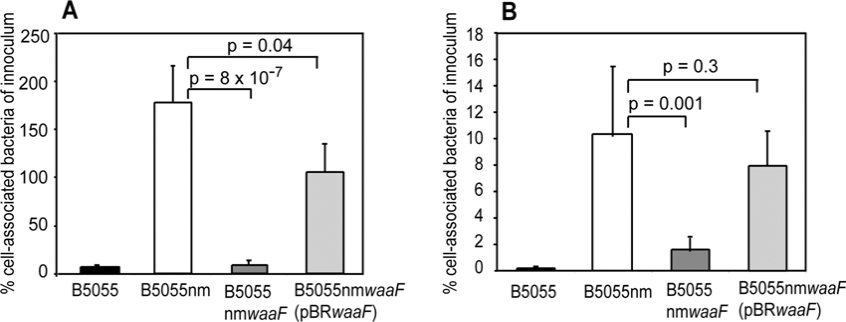
Association of *K. pneumoniae* mutant strains to mammalian cells. Bacteria were cultured to mid-log growth phase, washed, and added to confluent monolayers of RAW264.7 macrophage-like cells (A) or A549 epithelial cells (B). After 2 hrs, non-adherent bacteria were removed by repeated washing, cells were treated with Triton X-100 and cell lysates were plated for CFU enumeration. The number of cell associated bacteria are presented as the percentage of the initial inoculum. The data are the mean and standard deviation (SD) of 6 wells/strain and the assay was repeated at least three times.

As expected, the unencapsulated mutant B5055nm showed increased cell association (greater than 10-fold) to both cell lines compared with the wild type capsulated strain, confirming previous findings that the capsule reduces bacterial adherence [31], [33] (Fig. 2a and b). Although there was much higher cell-association overall to RAW cells (likely due to the phagocytosis/internalisation of bacteria by this macrophage-like cell line), the relative increase in cell-association that occurred following removal of the capsule was similar in the two cell types.

If the increased cell-association of the unencapsulated B5055nm was due simply to exposure of short proteinaceous adhesins, we hypothesised that bacteria lacking both LPS and capsular exopolysaccharides would show further increased cell association. Instead B5055nm*waaF* showed decreased cell-association compared with B5055nm. This decrease was approximately 5-fold for A549 cells and approximately 10-fold for RAW cells (Fig. 2a and b). The wild type level of association was restored in the complemented strains confirming the effect was due to the absence of the LPS polysaccharides rather than a secondary mutation. Cell-association experiments with A549 cells were repeated omitting the centrifugation step with no alteration of results.

The phenomonen of bacterial adhesion can be separated into 2 major processes; long range electrostatic interactions that allow a bacterium to come into contact with the cell/substrate etc; and short range specific adhesin interactions. Therefore we investigated whether the decreased cell-association of B5055nm*waaF* was a direct consequence of the loss of an LPS adhesin, ie. whether the O-Ag could act as an adhesin per se, or whether the reduced cell-association was an indirect effect resulting from altered electrochemical properties, affecting long-range interactions between the bacteria and the eukaryotic cell membrane.

### O-Ag of LPS as an adhesin

Two methods were utilised to determine whether LPS saccharides were directly acting as an adhesin. Firstly purified LPS, either intact or saccharides alone (acetic acid treated) were used to competitively inhibit LPS-mediated association with cells [34]. If specific LPS-A549 interactions were occurring free LPS molecules should adhere to their specific A549 receptors reducing the available binding sites for cell-bound LPS, and hence reducing the final cell-associated bacterial count. Secondly Fab fragments of the mAb I12, which specifically binds to the O1-Ag of LPS, were used to coat LPS molecules on the bacterial cell surface prior to addition of the bacteria to the cell association assay in order to prevent LPS-mediated cell association. No difference in bacterial association was observed with either treatment suggesting that the LPS saccharides were not specifically acting as an adhesin for A549 cells.

### Electrohydrodynamic characteristics of the bacterial strains

**Figure 3:**
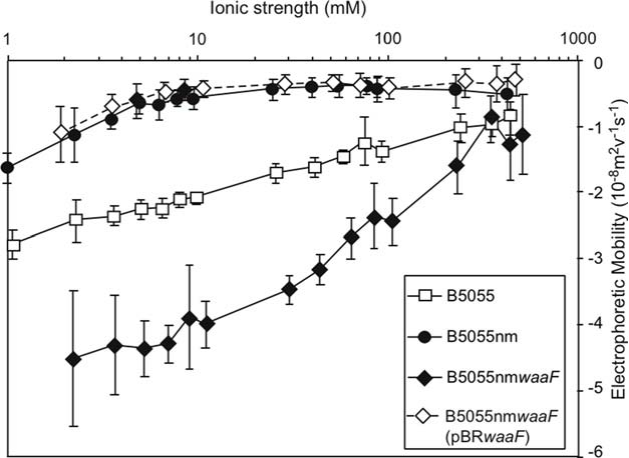
*K. pneumoniae* CPS and LPS saccharides affect the electrophoretic mobility of the bacteria. The electrophoretic mobility of B5055, B5055nm, B5055nm*waaF* and B5055nm*waaF* (pBR*waaF*) were compared in different ionic strength potassium nitrate buffers at the natural pH of the buffer (pH 5.5–6). Shown are the mean and SD of 60 trajectories for each strain, under each condition. The assay was repeated on different days with fresh cultures.

The electrophoretic mobilities of B5055, B5055nm, B5055nm*waaF* and B5055nm*waaF* (pBR*waaF*) were measured over a broad range of ionic strengths (Fig. 3). In general, the mobilities of the bacteria decreased (in absolute value) when the ionic strength of the buffer increased. This was due to masking of the bacterial charge by the mobile electrolyte ions, as anticipated from classical electric double layer theory. These results indicate that the nature of the bacterial surface ultrastructure impacts significantly on the magnitude of the mobility of the bacterium, a phenomenon that is dependent on the ionic environment. The electrophoretic mobilities of bacteria which present LPS O1-Ag as the outermost layer (ie. B5055nm) were significantly lower than the mobilities of bacteria devoid of O-Ag (ie. B5055nm*waaF*). The ionic strength-mobility patterns were also significantly different for these two strains. The presence of K2 capsule for the wild type encapsulated B5055 led to an intermediate electrokinetic response as compared to that for B5055nm and B5055nm*waaF*. The complemented strain, B5055nm*waaF* (pBR*waaF*) fully recovered the electrokinetic features of the unencapsulated mutant (B5055nm).

**Figure 4:**
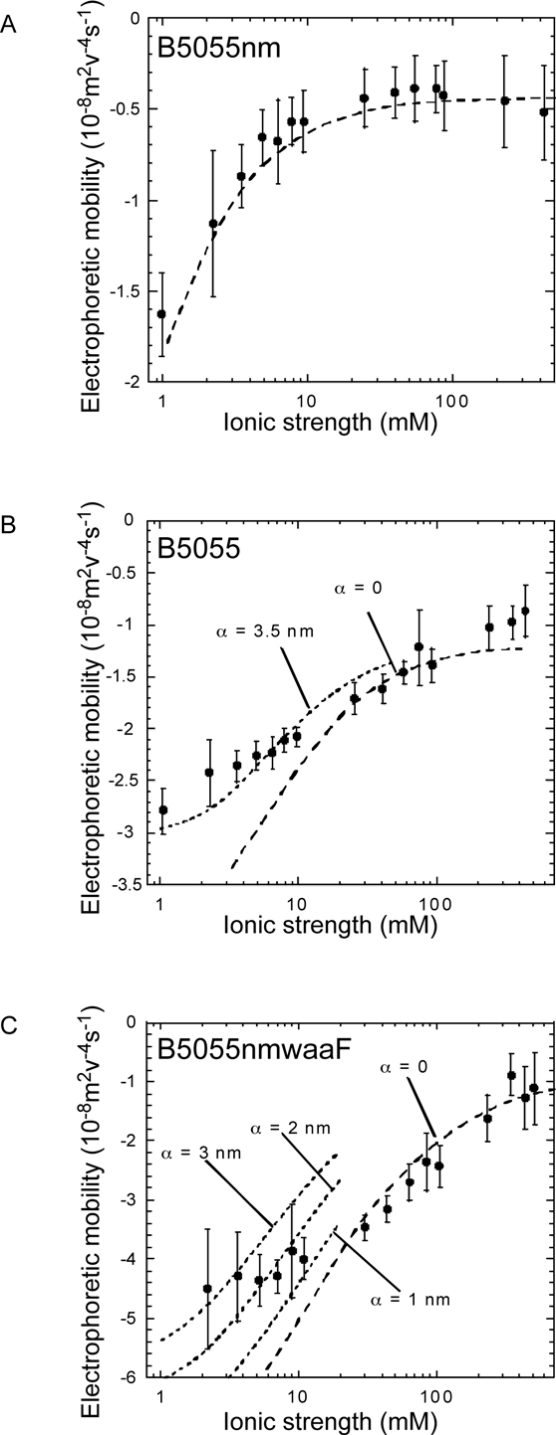
Theoretical description of the experimental electrophoretic mobilities of *K. pneumoniae* strains. The electrophoretic mobility (filled circles) are graphed for A) B5055nm, B) B5055 and C) B5055nm*waaF K. pneumoniae* strains expressed as a function of ionic strength. The calculations were performed either by using a homogeneous description for the spatial distribution of the soft-permeable polymer (α = 0, dash lines) or using a heterogeneous distribution (α>0, dotted lines) to reproduce the experimental data over the whole range of ionic strength considered. The different parameters obtained from the best fitting procedure are indicated in Table 1.

Theoretical computations were performed to quantitatively evaluate the electrohydrodynamic characteristics of the bacterial interface. These calculations were used to account for the experimental data and in particular, evaluate the volume charge density (*ρ*_o_) and the flow penetration degree (*λ*_o_^−1^) of the “soft” layer (the surface ultrastructure) surrounding the bacteria, namely the capsule for B5055 (160 nm thick), the LPS O1-Ag layer for B5055nm (30 nm thick) and the outer membrane for B5055nm*waaF* (2 nm thick). The resulting best-fit calculations are shown in Fig. 4 and the relevant parameters extracted from such analysis are collected in Table 2.

**Table 2:** Relevant electrokinetic parameters extracted from the soft particle analysis.

Strain	ρ_o_ (mM)	λ_o_^−1^ (nm)	α (nm)
B5055nm	−2.8	3.85	0
B5055	−22	2.3	3.5
B5055nmwaaF	−180	0.75	1–3

*ρ*_o_ = volume charge density (or electrochemical charge).

*λ*_o_^−1^ = flow penetration degree.

*α* = degree of heterogeneity (or diffuseness) for the spatial distribution of the permeable-soft layer.

Quantitative evaluation of electrophoretic mobility of the different strains allowed dissection of the electrostatic and hydrodynamic interfacial properties of the bacteria [35]–[38]. The electrophoretic mobility for B5055 and B5055nm was governed by the physicochemical characteristics (electrostatic and hydrodynamic) of the K2 capsule and LPS O1-Ag surface elements respectively, as the thickness of those two layers exceed, by at least an order of magnitude, the determined typical hydrodynamic penetration lengths *λ*_o_^−1^. Therefore, the analysis of the wild type and modified bacterial strains enabled independent evaluations of the electrokinetic features of these surface structures (capsule and LPS). The unencapsulated B5055nm strain demonstrated significantly lower (1/10th) volume charge density and somewhat higher hydrodynamic permeability compared to the encapsulated B5055 strain. This suggested that the electrical charge resulting from the very permeable LPS O1-Ag was significantly lower than that from the K2 capsule and that although the K2 capsule layer may be considered “soft” or permeable, the flow penetration into the inner LPS O1- Ag surface element is more significant than that into a K2 capsule layer. These results suggest that (i) the glucuronic acid residue in the K2 capsule is responsible for the volume charge density in B5055 and (ii) the thick K2 capsule provides a hydrodynamic barrier that separates the inner bacterial material from the outer electrolyte solution.

B5055nm*waaF*, which lacks the O1-Ag surface layer and the K2 capsule, exhibited the largest volume charge density and the lowest hydrodynamic permeability degree among the three analysed bacterial strains. This was observed because electro-osmotic flow probes a large number of charges (negatively charged core saccharides (GalA, KDO) and outer membrane proteins) distributed within a confined volume layer (2 nm thick) located in close proximity to the impermeable core of the bacterial surface structure (i.e. the region where the friction exerted on fluid flow is larger or, where the local hydrodynamic permeability is lower). As outlined in previous analyses [39], the heterogeneity of the interface is intrinsically related to the magnitude of the charge density carried by the soft, flexible (deformable) part of the bacteria, and to the hydrodynamic permeability of the bioparticle (as determined by local steric hindrance to flow). In more detail, the larger the *ρ*_o_ and the lower the permeability, the larger the interfacial osmotic pressure drop and the more heterogeneous the polymer segment density distribution from the rigid core to the outer periphery of the bacteria as a result of anisotropic water uptake within the periphery, and the soft central component of the bacteria. These theoretical trends are exactly those obtained for the three bacterial strains examined here (see Table 2).

## Discussion

Bacteria are constantly adapting to changing environments and must balance any adaptations with the need to conserve energy output. Therefore, high energy-requiring processes are likely to be essential for bacterial survival in selected environments. The production of both capsule and LPS are complex processes, requiring high energy expenditure, and both structures are therefore likely to be essential for a bacterium's survival and/or virulence. Both capsule and LPS appear to be constitutively expressed at a basal level but can be upregulated and modified where this is advantageous to survival of the organism [40]–[42].

Previous work has suggested that the *K. pneumoniae* capsule is necessary for resistance to phagocytosis while the O-Ag is essential for resistance to complement mediated lysis [27], [28], [43]. In the current study however, we found that the capsule but not LPS was essential for resistance to complement-mediated lysis. Confirmation that the O1-Ag was present on the cell surface by FACS analysis demonstrated that the O1-Ag saccharides alone on the cell surface were unable to protect against lysis by complement, further validating findings that the capsule modulates C3 deposition [26]. Our results confirmed the necessity of capsule production for *K. pneumoniae* survival and virulence in mammalian hosts but raised questions as to the role of the LPS saccharides if they were not involved in resistance to complement mediated lysis. The importance of the O-Ag to *K. pneumoniae* is inferred by its presence in over 90% of clinical isolates, the most common serotype of which is O1 [16], [44], suggesting an advantage to producing this serotype over other O types.

While investigating the ability of strains to be phagocytosed by a murine macrophage-like cell line (RAW264.7), we observed that B5055nm*waaF*, the strain lacking both capsule and LPS, had decreased association with these cells compared to the unencapsulated but O1-Ag positive strain, B5055nm. The increased cell-association of unencapsulated B5055nm compared to encapsulated B5055 has previously been attributed to the ability of the capsule to physically shield short adhesins [31], [32]. As such, it would be expected that further exposing these adhesins by removal of the LPS saccharide layer would again increase adherence. However, in this study we found that LPS contributed to long range electrostatic interactions rather than short range adhesin-specific processes. Investigation of the volume charge density of these strains confirmed a high negative charge density on the surface of B5055nm*waaF*, which we propose interfered with long range interactions by significantly increasing the repulsive electrostatic forces between the eukaryotic and bacterial cells. This would lead to decreased association of B5055nm*waaF* with eukaryotic cells, regardless of the presence of specific adhesins since the bacteria and eukaryotic cells would not be in close enough proximity to allow specific interactions to be engaged.

From these studies we conclude that one function of the O-Ag of *K. pneumoniae* involves presentation of a neutrally charged surface to the host. This supports previous hypotheses by Walker *et al* that long uncharged O-Ags of *E. coli* K12 shielded the charged groups of the bacterial membrane thus increasing bacterial interaction with quartz surfaces [45]. One outcome of this charge ‘masking’ would be to overcome long-range repulsion between bacteria and eukaryotic cells allowing specific adherence to occur. In addition, there are likely to be other consequences of this function such as a reduction in binding of charged particles, eg. antimicrobial peptides, to the bacterial cell surface. The lung defensin SP-D interacts with core LPS sugars but has been shown to be ineffective against O1-Ag positive *K. pneumoniae* strains [46]. This may result in part from steric hindrance, however the inability to detect the charged core region due to the presence of neutral O1-Ag may also contribute to this reduced efficacy. Therefore although we have demonstrated one important outcome of removing the neutral O1-Ag charge, namely decreased cell association, there may be other consequences which would make expression of the neutral O1-Ag advantageous for virulence in *K. pneumoniae*.

## Methods

### Bacterial strains and plasmids

The *K. pneumoniae* strain used was B5055, a mouse virulent clinical isolate (serotype K2:O1) and a non-encapsulated mutant of B5055 designated B5055nm, produced by Dr T. Uren and Dr A. Jenney (Department of Microbiology and Immunology, University of Melbourne) through the inactivation of *wzb* and *wzc*. Briefly a 1.1 kb HindIII fragment spanning *wzb* and *wzc* was removed and a 1.6 kb HindIII fragment from pUC4KIXX containing the kanamycin resistance cassette inserted. Genomic recombination was achieved by electroporating the linear wzb-kan-wzc fragment into B5055 containing induced λ red recombinase and selecting for kanamycin resistant mutants. *E. coli* JM109 and the plasmids pGEM T-Easy or pBR322 (Promega) were used for cloning experiments. The temperature sensitive plasmid pKD46 [47] was used to induce λ red recombinase production in the host cell in order to enhance homologous recombination. Bacteria were grown in Luria broth (LB) or on Luria agar (LA) at 37°C (except for induction of λ red recombinase when bacteria were cultured at 30°C). Antibiotics were used at the following concentrations: ampicillin at 100 µg/ml, kanamycin at 60 µg/ml and chloramphenicol at 30 µg/ml.

### Generation of K. pneumoniae *waaF* mutant

The *waa* operon of B5055 was sequenced and determined to be of the core 2 serotype [48]. The second gene in the operon, the *waaF* gene (plus 500 bp at either end of the open reading frame) was amplified from B5055 using oligonucleotide primers (5′-GGAAGCCAATTCGCAGATCG-3′ and 5′-GCTTTTGGCGAACAGCTCAC-3′) and cloned into pGEM T-Easy (to form pGEM-*waaF*), followed by digestion with XmaI and StuI to remove a central 362 bp fragment. A chloramphenicol resistance cassette (*cat*) from pACYC184 was amplified with primers 5′-GATCACCCGGGGTAGCACCAGGCGTTTAAGGG-3′ and 5′-ACTGTAGGCCTCGTAAGAGGTTCCAACTTTCAC-3′ and cloned into pGEM-*waaF* using the underlined XmaI and StuI sites (to form pGEM-*waaF*/Chl). pKD46 was electroporated into B5055 (2.5 kV, 25 µF, 200 Ω) or B5055nm (1.8 kV, 25 µF, 200 Ω) and grown at 30°C with L-arabinose to induce λ red recombinase production. The *waaF*-Chl fragment was then amplified as a 2.5 kb linear fragment, gel purified and digested with NotI to remove any residual intact plasmid. The linear DNA fragment was electroporated into B5055+pKD46 or B5055nm+pKD46 and grown at 37°C on chloramphenicol to remove pKD46 and select for recombinants respectively. The anticipated disrupted *waaF* gene size was confirmed by PCR and confirmed recombinants designated B5055*waaF* and B5055nm*waaF* (for capsulated and unencapsulated strains respectively). Complementation was achieved with two constructs pGEM-*waaoperon* (where the entire waa operon, including the native promoter was inserted into pGEM T-EASY) and pBR*waaF* (where the *waaF* open reading frame was inserted such that it was transcribed from the *lacZ* promoter of pGEM, and translated from the native AUG start codon using the cognate *waaF* ribosome binding site and then amplified with 5′-CCGGAATTCGTCCATTCGCCATTCAGG-3′ and 5′- CCGGGATCCGAGCGAGGAAGCGGAAGAG-3′ and inserted into pBR322). Not all mutants were able to be complemented and only strains that could be complemented were used for further studies.

### Monoclonal antibody preparation

The monoclonal antibodies C13 and I12 were produced following immunisation of BALB/c mice with the sarkosyl insoluble outer membrane protein fraction of B5055nm. The spleen cells from these immunised animals were fused with non-secretory BALB/c myeloma Sp2/0-Ag14 using 50% w/v polyethylene glycol (Sigma Hybridoma Starter Pack, Sigma, Castle Hill, NSW Australia) essentially as described previously [49]. Following fusion, screening and sub-cloning, hybridoma supernatants were purified by protein G sepharose columns (Sigma). C13 and I12 were determined to bind to the O1-Ag of B5055 by western blot and ELISA against purified LPS (results not shown). FAb fragments of I12 were produced by digestion on immobilised papain (Pierce) and purification on a protein A sepharose column (Pierce).

### SDS-PAGE analysis of LPS

Crude LPS preparations were made by pelleting cells from 3 ml of overnight (o/n) cultures. The pellets were resuspended in Laemmli buffer (2.3% SDS, 0.8% Tris, 10% glycerol, 5% DTT) and samples boiled for 10 minutes. Samples were digested with proteinase K (0.417 µg/µl) for 2 h at 56°C. SDS-PAGE was performed on 15% polyacrylamide gels [50] and stained with alkaline silver stain to visualize LPS [23].

### Purification of LPS

LPS was purified by modified hot phenol extraction [51]. Stationary phase cultures (500 ml) were pelleted and washed with PBS. The bacterial pellet was resuspended in a small volume of PBS and cells disrupted by sonication. Debris was removed by centrifugation at 5,400×g for 45 min and the supernatant centrifuged for 1 hr at 16,000×g. The resulting pellet was resuspended in 500 µl of 2 mM Tris-HCl, pH 7.8 and treated with DNase and RNase for 2 hr at 37°C followed by Proteinase K treatment for 2 hr at 56°C. Samples were heated to 65°C, mixed 1:1 with pre-heated phenol and incubated for 15 min at 65°C. Samples were then cooled to room temperature and centrifuged to separate the phases. The aqueous phase containing purified LPS was removed and the phenol phase re-extracted with water.

The lipid A was cleaved from the LPS preparation by 1% acetic acid treatment at 100°C and removed by centrifugation. All LPS preparations were dialysed against water or PBS prior to use.

### Extraction and quantification of capsule

Cell associated capsule was extracted from mid-log and static cultures by the phenol-extraction method [51]. Briefly, 5 ml samples were centrifuged (5000 g, 15 min, 4°C), washed, resuspended in 500 µl dH_2_O and viable counts determined. Samples were then incubated at 68°C for 2 min before addition of 500 µl phenol, incubation was then continued for 30 min. The mixture was cooled and 500 µl chloroform added, centrifuged and the aqueous phase collected. Capsular material was precipitated at −20°C for 20 min and resuspended in 500 µl dH_2_O.

Uronic acid content was determined as previously described [52]. Briefly, 1.2 ml of 12.5 mM tetraborate in concentrated H_2_SO_4_ was added to 200 µl sample and vortexed vigorously. Samples were then boiled for 5 min and cooled before addition of 20 µl 0.15% 3-hydroxydiphenol in 0.5% NaOH. Absorbance was measured at 520 nm. Standard curves were constructed with glucuronic acid.

Bacterial capsules were also visualized by Maneval's stain [53].

### OMP preparation and analysis

OMPs were prepared from mid-log cultures by sarkosyl separation [54]. Brifely bacteria were grown to mid-log (2.5 hrs with shaking), pelleted, resuspended in 10 mM HEPES and disrupted by sonication. Unbroken cells were removed by centrifugation and membrane proteins (inner and outer membranes) pelleted by ultracentrifugation (150,000 g, 60 min, 4°C). Inner membrane proteins were solubilized in 1% N-lauroylsarcosine and insoluble outer membrane proteins collected by ultracentrifugation (120,000 g, 40 min, 4°C).

OMPs were then analysed on 12.5% SDS-PAGE followed by coomassie staining (20 µg protein per lane) or western blot (5 µg protein per lane).

### Cell association Assay

RAW 264.7 (murine macrophage-like) or A549 (human lung epithelial type II) cells were seeded at ∼2.5×10^5^cells/well in a 24 well tissue culture plate (Nalge Nunc International) and grown to confluence (approximately 18 h) at 37°C, 5% CO_2_. Bacteria grown to mid-log phase at 37°C with shaking were washed once with PBS and resuspended in RPMI or DMEM (without supplements) as appropriate. Tissue culture cells were washed once with fresh media and 500 µl of the appropriate supplemented media added. 100 µl of undiluted bacterial suspension was added to each well and a sample retained to determine the inoculation dose. Plates were centrifuged at 500×g for 5 min to increase interaction of bacteria and cells and incubated for 2 h at 37°C, 5% CO_2_. Wells were washed three times with fresh media and 100 µl 0.1% Triton X-100 added for 10 min at RT to lyse the eukaryote cell membranes. 400 µl LB was added to the wells and the number of viable bacteria determined by culturing on selective media. Results were expressed as mean and standard variation and statistical difference assessed by unpaired two-tailed student t-test.

### Electrophoretic mobility measurements

Bacteria were grown sequentially on LA o/n, in LB o/n and finally to mid-log in 50 ml LB. Bacteria were pelleted (10 min, 10,000×g), washed once with 1 mM KNO_3_ and resuspended in 1 mM KNO_3_ at ∼10^7^ cfu/ml. Electrophoretic mobility measurements were then performed (Zetaphoremeter IV, CAD Instrumentations, Les Essarts le Roi, France) in a quartz suprasil cell at room temperature (22–24°C). The bacterial mobilities were measured by reflection of a laser beam and tracked with a charge-coupled device camera. Image analysis software allowed recorded images to be processed in real time to calculate the electrophoretic mobilities from the displacement of bacteria subjected to a constant direct-current electric field (800 V/m). Different cycles were performed and 60 mean bacterial trajectory was obtained for each condition. All the experiments were carried out in natural pH (∼6) and repeated twice with different cultures.

### Soft particle analysis of the electrophoretic mobilities

The dependence of the electrophoretic mobilities on the ionic strength was analysed as recently described (Duval et al. 2006). Briefly, the electrokinetic response of the bacteria was assimilated to that of a soft particle composed of an impermeable hard-core component covered with a permeable polyelectrolyte layer. The bacterial cells were modelled as soft spherical entities (microscopic analysis indicated this strain grew as short bacilli, more closely resembling a sphere than a rod-shape) of core radius equal to 1 µm and the thickness (*δ*) of the permeable layer was estimated at 160 nm for B5055 [55], 30 nm for the B5055nm [8], [9] and 2 nm for the B5055nm*waa*F strain [56]. The experimental data were fitted using the method of least mean squares to determine (i) the permeability degree of the bacteria, as reflected by the typical length (*λ*_o_^−1^) of flow permeation within the charged polyelectrolyte layer, (ii) the volume charge density (*ρ*_o_) of the soft permeable component of the bacteria and (iii) the degree of heterogeneity/diffuseness (subsumed in a parameter denoted as α) for the charged polymer segment density distribution within the soft layer. For sufficiently high electrolyte concentrations and/or low volume charge layer densities, the electrohydrodynamic features of the bacteria are essentially independent of the spatial distribution of the soft material density at the bacteria/aqueous solution interface, basically converting to homogeneous step-function like bacterial interfaces (*α* = 0). This peculiarity allows a practical determination of the parameter (*λ*_o_^−1^) and the volume charge density (*ρ*_o_). Any deviations of theoretical predictions from the experimental data (eg. at low ionic strengths) were quantitatively interpreted by adjustment of the diffuse character (*α*) of the interface bacteria/electrolyte solution. This increase of interfacial heterogeneity with lowering electrolyte concentration is the result of swelling processes partly due to large intramolecular repulsive forces within the polymer.

## References

[1] Moses AE, Wessels MR, Zalcman K, Alberti S, Natanson-Yaron S (1997). Relative contributions of hyaluronic acid capsule and M protein to virulence in a mucoid strain of the group A Streptococcus.. Infect Immun.

[2] Vogel U, Hammerschmidt S, Frosch M (1996). Sialic acids of both the capsule and the sialylated lipooligosaccharide of Neisseria meningitis serogroup B are prerequisites for virulence of meningococci in the infant rat.. Med Microbiol Immunol.

[3] Danese PN, Pratt LA, Kolter R (2000). Exopolysaccharide production is required for development of Escherichia coli K-12 biofilm architecture.. J Bacteriol.

[4] Costerton JW, Lewandowski Z, Caldwell DE, Korber DR, Lappin-Scott HM (1995). Microbial biofilms.. Annu Rev Microbiol.

[5] Okamoto S, Kawabata S, Terao Y, Fujitaka H, Okuno Y (2004). The Streptococcus pyogenes capsule is required for adhesion of bacteria to virus-infected alveolar epithelial cells and lethal bacterial-viral superinfection.. Infect Immun.

[6] Ophir T, Gutnick DL (1994). A Role for Exopolysaccharides in the Protection of Microorganisms from Desiccation.. Appl Environ Microbiol.

[7] Alexander C, Rietschel ET (2001). Bacterial lipopolysaccharides and innate immunity.. J Endotoxin Res.

[8] Kastowsky M, Gutberlet T, Bradaczek H (1992). Molecular modelling of the three-dimensional structure and conformational flexibility of bacterial lipopolysaccharide.. J Bacteriol.

[9] Burks GA, Velegol SB, Paramonova E, Lindenmuth BE, Feick JD (2003). Macroscopic and Nanoscale Measurements of the Adhesion of Bacteria with Varying Outer Layer Surface Composition.. Langmuir.

[10] Rosenthal S, Tager IB (1975). Prevalence of gram-negative rods in the normal pharyngeal flora.. Ann Intern Med.

[11] Johanson WG, Pierce AK, Sanford JP (1969). Changing pharyngeal bacterial flora of hospitalized patients. Emergence of gram-negative bacilli.. N Engl J Med.

[12] Waters V, Larson E, Wu F, San Gabriel P, Haas J (2004). Molecular epidemiology of gram-negative bacilli from infected neonates and health care workers' hands in neonatal intensive care units.. Clin Infect Dis.

[13] Neuhauser MM, Weinstein RA, Rydman R, Danziger LH, Karam G (2003). Antibiotic resistance among gram-negative bacilli in US intensive care units: implications for fluoroquinolone use.. Jama.

[14] Hanberger H, Garcia-Rodriguez JA, Gobernado M, Goossens H, Nilsson LE (1999). Antibiotic susceptibility among aerobic gram-negative bacilli in intensive care units in 5 European countries. French and Portuguese ICU Study Groups.. Jama.

[15] Couto RC, Carvalho EA, Pedrosa TM, Pedroso ER, Neto MC (2007). A 10-year prospective surveillance of nosocomial infections in neonatal intensive care units.. Am J Infect Control.

[16] Hansen DS, Mestre F, Alberti S, Hernandez-Alles S, Alvarez D (1999). Klebsiella pneumoniae lipopolysaccharide O typing: revision of prototype strains and O-group distribution among clinical isolates from different sources and countries.. J Clin Microbiol.

[17] Fung CP, Hu BS, Chang FY, Lee SC, Kuo BI (2000). A 5-year study of the seroepidemiology of Klebsiella pneumoniae: high prevalence of capsular serotype K1 in Taiwan and implication for vaccine efficacy.. J Infect Dis.

[18] Jenney AW, Clements A, Farn JL, Wijburg OL, McGlinchey A (2006). Seroepidemiology of Klebsiella pneumoniae in an Australian Tertiary Hospital and its implications for vaccine development.. J Clin Microbiol.

[19] Corsaro MM, De Castro C, Naldi T, Parrilli M, Tomas JM (2005). 1H and 13C NMR characterization and secondary structure of the K2 polysaccharide of Klebsiella pneumoniae strain 52145.. Carbohydr Res.

[20] Vinogradov E, Frirdich E, MacLean LL, Perry MB, Petersen BO (2002). Structures of lipopolysaccharides from Klebsiella pneumoniae. Eluicidation of the structure of the linkage region between core and polysaccharide O chain and identification of the residues at the non-reducing termini of the O chains.. J Biol Chem.

[21] Frirdich E, Bouwman C, Vinogradov E, Whitfield C (2005). The role of galacturonic acid in outer membrane stability in Klebsiella pneumoniae.. J Biol Chem.

[22] Fresno S, Jimenez N, Canals R, Merino S, Corsaro MM (2007). A Second Galacturonic Acid Transferase Is Required for Core Lipopolysaccharide Biosynthesis and Complete Capsule Association with the Cell Surface in Klebsiella pneumoniae.. J Bacteriol.

[23] Tsai CM, Frasch CE (1982). A sensitive silver stain for detecting lipopolysaccharides in polyacrylamide gels.. Anal Biochem.

[24] Ried G, Hindennach I, Henning U (1990). Role of lipopolysaccharide in assembly of Escherichia coli outer membrane proteins OmpA, OmpC, and OmpF.. J Bacteriol.

[25] Domenico P, Tomas JM, Merino S, Rubires X, Cunha BA (1999). Surface antigen exposure by bismuth dimercaprol suppression of Klebsiella pneumoniae capsular polysaccharide.. Infect Immun.

[26] Cortes G, Borrell N, de Astorza B, Gomez C, Sauleda J (2002). Molecular analysis of the contribution of the capsular polysaccharide and the lipopolysaccharide O side chain to the virulence of Klebsiella pneumoniae in a murine model of pneumonia.. Infect Immun.

[27] Merino S, Camprubi S, Alberti S, Benedi VJ, Tomas JM (1992). Mechanisms of Klebsiella pneumoniae resistance to complement-mediated killing.. Infect Immun.

[28] Tomas JM, Benedi VJ, Ciurana B, Jofre J (1986). Role of capsule and O antigen in resistance of Klebsiella pneumoniae to serum bactericidal activity.. Infect Immun.

[29] Clements A, Tull D, Jenney AW, Farn JL, Kim SH (2007). Secondary acylation of Klebsiella pneumoniae lipopolysaccharide contributes to sensitivity to antibacterial peptides.. J Biol Chem.

[30] Pizarro-Cerda J, Cossart P (2006). Bacterial adhesion and entry into host cells.. Cell.

[31] Schembri MA, Dalsgaard D, Klemm P (2004). Capsule shields the function of short bacterial adhesins.. J Bacteriol.

[32] Schembri MA, Blom J, Krogfelt KA, Klemm P (2005). Capsule and fimbria interaction in Klebsiella pneumoniae.. Infect Immun.

[33] Sahly H, Podschun R, Oelschlaeger TA, Greiwe M, Parolis H (2000). Capsule impedes adhesion to and invasion of epithelial cells by Klebsiella pneumoniae.. Infect Immun.

[34] Belanger M, Dubreuil D, Harel J, Girard C, Jacques M (1990). Role of lipopolysaccharides in adherence of Actinobacillus pleuropneumoniae to porcine tracheal rings.. Infect Immun.

[35] Duval JFL, Ohshima H (2006). Electrophoresis of diffuse soft particles.. Langmuir.

[36] Dague E, Duval JFL, Jorand F, Thomas F, Gaboriaud F (2006). Probing surface structures of Shewanella spp. by microelectrophoresis.. Biophys J.

[37] Duval JFL, Busscher HJ, van de Belt-Gritter B, van der Mei HC, Norde W (2005). Analysis of the interfacial properties of fibrillated and nonfibrillated oral streptococcal strains from electrophoretic mobility and titration measurements: evidence for the shortcomings of the ‘classical soft-particle approach’.. Langmuir.

[38] Gaboriaud F, Gee ML, Strugnell R, Duval JFL (2008). Coupled electrostatic, hydrodynamic, and mechanical properties of bacterial interfaces in aqueous media.. Langmuir.

[39] Rotureau E, Thomas F, Duval JFL (2007). Relationship between swelling and the electrohydrodynamic properties of functionalized carboxymethyldextran macromolecules.. Langmuir.

[40] Lai YC, Peng HL, Chang HY (2003). RmpA2, an activator of capsule biosynthesis in Klebsiella pneumoniae CG43, regulates K2 cps gene expression at the transcriptional level.. J Bacteriol.

[41] Wehland M, Bernhard F (2000). The RcsAB box. Characterization of a new operator essential for the regulation of exopolysaccharide biosynthesis in enteric bacteria.. J Biol Chem.

[42] Whitfield C, Roberts IS (1999). Structure, assembly and regulation of expression of capsules in Escherichia coli.. Mol Microbiol.

[43] Kabha K, Nissimov L, Athamna A, Keisari Y, Parolis H (1995). Relationships among capsular structure, phagocytosis, and mouse virulence in Klebsiella pneumoniae.. Infect Immun.

[44] Trautmann M, Held TK, Cross AS (2004). O antigen seroepidemiology of Klebsiella clinical isolates and implications for immunoprophylaxis of Klebsiella infections.. Vaccine.

[45] Walker SL, Redman JA, Elimelech M (2004). Role of Cell Surface Lipopolysaccharides in Escherichia coli K12 adhesion and transport.. Langmuir.

[46] Kostina E, Ofek I, Crouch E, Friedman R, Sirota L (2005). Noncapsulated Klebsiella pneumoniae bearing mannose-containing O antigens is rapidly eradicated from mouse lung and triggers cytokine production by macrophages following opsonization with surfactant protein D.. Infect Immun.

[47] Datsenko KA, Wanner BL (2000). One-step inactivation of chromosomal genes in Escherichia coli K-12 using PCR products.. Proc Natl Acad Sci U S A.

[48] Regue M, Izquierdo L, Fresno S, Pique N, Corsaro MM (2005). A second outer-core region in Klebsiella pneumoniae lipopolysaccharide.. J Bacteriol.

[49] Adler B, Faine S (1983). A pomona serogroup-specific, agglutinating antigen in Leptospira, identified by monoclonal antibodies.. Pathology.

[50] Laemmli UK (1970). Cleavage of structural proteins during the assembly of the head of bacteriophage T4.. Nature.

[51] Westphal O, Jann K (1963). Bacterial lipopolysaccharides extraction with phenol-water and further applications of the procedure.. Methods Carbohydrate Chemistry.

[52] Blumenkrantz N, Asboe-Hansen G (1973). New method for quantitative determination of uronic acids.. Anal Biochem.

[53] Maneval WE (1941). Staining bacteria and yeasts with acid dyes.. Staining technology.

[54] Filip C, Fletcher G, Wulff JL, Earhart CF (1973). Solubilization of the cytoplasmic membrane of Escherichia coli by the ionic detergent sodium-lauryl sarcosinate.. J Bacteriol.

[55] Amako K, Meno Y, Takade A (1988). Fine structures of the capsules of Klebsiella pneumoniae and Escherichia coli K1.. J Bacteriol.

[56] Beveridge TJ (1999). Structures of gram-negative cell walls and their derived membrane vesicles.. J Bacteriol.

